# Experimental insight into the proximate causes of male persistence variation among two strains of the androdioecious *Caenorhabditis elegans *(Nematoda)

**DOI:** 10.1186/1472-6785-8-12

**Published:** 2008-07-13

**Authors:** Viktoria Wegewitz, Hinrich Schulenburg, Adrian Streit

**Affiliations:** 1 Department of Evolutionary Biology, Max Planck Institute for Developmental Biology, Tübingen, Germany; 2 Institute of Zoology, University of Tübingen, Tübingen, Germany

## Abstract

**Background:**

In the androdioecious nematode *Caenorhabditis elegans *virtually all progeny produced by hermaphrodite self-fertilization is hermaphrodite while 50% of the progeny that results from cross-fertilization by a male is male. In the standard laboratory wild type strain N2 males disappear rapidly from populations. This is not the case in some other wild type isolates of *C. elegans*, among them the Hawaiian strain CB4856.

**Results:**

We determined the kinetics of the loss of males over time for multiple population sizes and wild isolates and found significant differences. We performed systematic inter- and intra-strain crosses with N2 and CB4856 and show that the males and the hermaphrodites contribute to the difference in male maintenance between these two strains. In particular, CB4856 males obtained a higher number of successful copulations than N2 males and sired correspondingly more cross-progeny. On the other hand, N2 hermaphrodites produced a higher number of self-progeny, both when singly mated and when not mated.

**Conclusion:**

These two differences have the potential to explain the observed variation in male persistence, since they should lead to a predominance of self-progeny (and thus hermaphrodites) in N2 and, at the same time, a high proportion of cross-progeny (and thus the presence of males as well as hermaphrodites) in CB4856.

## Background

The nematode *Caenorhabditis elegans *is a facultative hermaphrodite that reproduces either by virtue of self-fertilization or cross-breeding with a male (androdioecious reproductive system). The hermaphrodites are somatically female but produce a limited number of sperm during their late larval development before switching to the production of eggs. The sperm is stored in the spermatheca and can be used to fertilize the newly formed eggs. Except for a very few males (around 0.2% in the standard laboratory strain N2) that arise spontaneously as the result of X chromosome non-disjunction, the entire self-progeny is hermaphroditic. Males can mate with hermaphrodites and give rise to 50% males in the cross-progeny. Cross-fertilization is not possible among hermaphrodites [1,2]. Male derived sperm is also stored in the spermatheca where it competes with the hermaphrodite's own sperm for the fertilization of the oocytes. Male sperm is usually larger and therefore has a competitive advantage over the hermaphrodite's sperm [3,4].

If hermaphrodites can reproduce by self-fertilization, males are superfluous. In fact, they could even represent a burden, decreasing individual fitness, in analogy with the two-fold cost of males in theories on the evolution of sex [5,6]. Therefore, the persistence of males represents an important puzzle for our understanding of *C. elegans *biology. Its explanation is expected to advance a more general insight into the evolution of androdioecy. To date, the function of *C. elegans *males has been addressed using two main approaches: (i) experimental evolution in the laboratory, and (ii) analysis of (male-dependent) outcrossing rates in wild populations.

The experimental evolution of laboratory populations of the standard strain N2 (as well as mutants derived from this strain) uniformly demonstrated that initial male frequencies of either 50% or 33% rapidly and steeply decline to less than 10% within ten to 15 generations [7-11]. Furthermore, compared with the dioecious species *Caenorhabditis remanei*, mating behaviour was severely compromised in the N2 strain, i.e. males often fail to find hermaphrodite mates, possibly due to limited production and/or degeneracy of the hermaphrodite's sex pheromone [[7,12] but see, [13,14]]. Taken together, these results suggested that males represent evolutionary relics without any particular function, which are only still present, because of a relatively recent switch to hermaphroditism and selfing in the lineage leading to *C. elegans *[7]. Interestingly, however, other natural isolates show clear differences to N2. The spontaneous production of males is a pre-requisite for male maintenance and it reaches values of more than 3% of the total offspring in some isolates – clearly more than N2 with a value of less than 0.5% [11,15]. Similarly, males are able to persist in populations of some strains, e.g. the Hawaiian strain CB4856 and the Oregon strain PX174 [11]. This effect seems to be enhanced in these two strains (but not others) if worm populations are subjected to fluctuating environmental conditions like variable exposure to different mutagens [16]. Similarly, populations with deficient DNA repair and thus increased mutations rates also maintain males at higher frequencies [10]. These results suggested that males are beneficial to ensure frequent outcrossing, which is favored under variable environmental conditions and/or high deleterious mutation rates [17-22].

An alternative albeit indirect route to assess the function of males is to infer outcrossing rates in natural populations. Several recent studies analysed new *C. elegans *isolates from different parts of the world using a variety of molecular markers such as microsatellites, AFLPs, or DNA sequence polymorphisms. They unanimously demonstrate that outcrossing does occur, but that it is usually extremely rare [23-27]. The only exception is an inferred outcrossing rate of 0.2 [28], whereas all other studies suggest it to range in between 10^-5 ^up to 0.02 [23-25]. Consequently, males leave a genetic footprint in natural populations. In consistency with the conclusions from experimental evolution in the lab, rare outcrossing may be sufficient to eliminate mutational load and/or maintain genetic diversity required for rapid adaptation to fluctuating environments [17,29,30].

In the first part of this publication we describe the decline of the proportion of males in eight different natural isolates under standard laboratory conditions. The fact that males are lost at very different rates even if the strains are maintained under the same conditions indicates that the difference is genetically determined and is therefore a putatively selectable trait. In the second part we evaluate the possible reasons for the difference in male persistence between the two common laboratory strains N2 and CB4856. Several behavioral and physiological factors could account for this difference, for example: i) the mating efficiency of the males, ii) the mating efficiency of the hermaphrodites (this includes the attractiveness of the hermaphrodites for males), iii) the competitive advantage of the male derived sperm, iv) the number of sperm transferred, v) the difference between the maximum number of progeny a hermaphrodite can produce with and without mating. In order to address these points, we performed a systematic analysis of intra- and inter-strain crosses between N2 and CB4856- to our knowledge for the very first time in this context. Our results indicate that CB4856 males are capable of mating successfully with more hermaphrodites than N2 males and that N2 hermaphrodites produced a higher number of (all-hermaphrodite) self-progeny even after mating. Both these effects result in a higher proportion of males in the next generation for CB4856 if compared with N2, thus potentially explaining male persistence in the former but not the latter strain.

## Methods

### C. elegans cultures

*C. elegans *was cultured as described in [31]. The preparation of NGM plates and *Escherichia. coli *strain OP50 food bacteria and M9 buffer is also described in [31].

Mating plates: 6 cm NGM plates were seeded with 30 μl of an *E. coli *(OP50) culture such that the plates contained a small round dot of bacteria in the center.

All experiments were done in an air-conditioned room at a temperature of 21 ± 1°C and 40% humidity. To minimize fluctuations of physical conditions plates were kept in boxes, randomized in piles that were placed evenly distributed within the boxes.

### Strains used

N2: Standard laboratory wild type strain, isolated in Bristol, UK

CB4856: Standard polymorphic mapping strain, isolated in Hawaii.

AB1: isolate from Australia

JU258: isolate from Madeira

MY1, MY15, MY18, RC301: isolates from Germany

All strains are available from the *Caenorhabditis *Genetics Center at the University of Minnesota [32].

### Male maintenance assay

This assay served to determine the persistence of males over time in different natural *C. elegans *isolates and in different population sizes. For each strain, we set up several crosses using a male:hermaphrodite ratio of 2:1. These crosses yielded populations with a gender ratio of approximately 1:1. For each strain, the populations were mixed four days after setting up the crosses and a defined number of individuals (population size) was randomly chosen and transferred onto NGM plates for the experiment (day 0 of the experiment). These experimental populations were all treated as follows: After three days adult males and hermaphrodites were counted (counting, see below). On the next day the population was reduced to the original population size and transferred to a new plate (transfer, see below). Three days later, adult males and hermaphrodites were counted again, followed by population size reduction and worm transfer one day later, as above. This whole procedure was repeated for a total of eight times (equivalent to 32 days). All male maintenance assays were done on 9 cm NGM plates, seeded with 1 ml of *E. coli *OP50 culture. Two sets of experiments were performed: i) two replica runs per strain and population size were done in parallel for strains N2, CB4856, AB1, JU258, MY1, MY15, MY18 and RC301 using population sizes of 75 and 150; and ii) five replica runs per strain and population size were done in parallel for N2 and CB4856 using population sizes of 40, 70, 100 and 150. In this context, one important objective was to evaluate the effect of different population sizes on male persistance. The exact numbers used (e.g. 75 versus 150) were chosen arbitrarily.

#### Counting

The plates were placed under a dissecting microscope and searched systematically always using the same search path with the help of a grid, which was positioned below the plates. The first 100 to 120 adults encountered were then used to determine the number of adult hermaphrodites and adult males. Note that this is equivalent to a random choice of individuals. Only hermaphrodites with developing embryos in the uterus were counted as adult hermaphrodites. This may have lead to a slight underestimation of the number of hermaphrodites.

#### Transfer

Worms were washed off plates with M9 buffer and counted without paying attention to the developmental stage or sex of the worms. The volume that was expected to contain the desired number of worms was transferred onto a new plate. Thus, the populations always consisted of mixed generations, so that the effective reproductive population was smaller than the actual number of animals.

### Male mating efficiency assay

This assay served to evaluate the mating efficiency of males in terms of mated hermaphrodites, total offspring produced per male and also cross- as well as self-progeny produced per mated hermaphrodite. A single male was confronted with an excess of hermaphrodites, so that it could mate as often as possible. In a pilot experiment, 14 hermaphrodites were found to be sufficient to ensure that the male would never come close to mating with all of them. In fact, during the main experiment the highest number of mated hermaphrodites per plate was 9.

Mating plates were prepared four days prior to the experiment. One male and 14 young adult hermaphrodites were placed on mating plates. Every 24 hours the male was moved to a new mating plate with 14 young adult hermaphrodites until no more successful mating was observed. Hermaphrodites, which were exposed to males, were placed individually on NGM plates seeded with 200 μl *E. coli *OP50. The hermaphrodites were transferred to new plates every 24 h. After three days the progeny was counted or, if the number of plates was too high to be processed immediately, equal numbers of plates from both treatments were put at 4°C and counted within a few days. This step allowed us to do more replicas in parallel and have all plates scored by the same person, in order to avoid possible observer biases. We did not observe any lethality in response to the cooling step. A hermaphrodite was considered to be mated when more than one male was found among the progeny (successful mating event). The number of cross progeny per mated hermaphrodite was estimated as twice the number of male progeny. Since it was not feasible to do all crosses in parallel, we used an incomplete block design, where two different crosses were set up in parallel and different pairs of parallel crosses were assayed in four experimental runs: i) N2 males crossed with N2 and with CB4856 hermaphrodites; ii) CB4856 males crossed with N2 and with CB4856 hermaphrodites; iii) N2 and CB4856 males crossed with N2 hermaphrodites; iv) N2 and CB4856 males crossed with CB4856 hermaphrodites.

### Mating behavior assays

These two assays were used to characterize in more detail the time required by males until first contact with a hermaphrodite and first spicule insertion (One-hour assay) and also the number of contacts with hermaphrodites as well as spicule insertions over a nine hour period (Nine-hours assay). In both assays, we used the same general conditions as in the male mating efficiency assay (particularly as to usage of 14 hermaphrodites), in order to permit comparison of results.

#### One hour assay

L4 males and L4 hermaphrodites were transferred to separate plates one day prior to testing in order to obtain virgin adult animals. At the beginning of the experiment, one male was placed together with 14 hermaphrodites on a mating plate. We then measured the time until the male touched a hermaphrodite and showed mating behavior (first contact) and until the first time the spicule was inserted (spicule insertion). Observations were terminated after spicule insertion or, if these did not occur, after one hour.

#### Nine-hours assay

14 L4 hermaphrodites per plate were placed on mating plates one day prior to the experiment. At the same time L4 males were collected and placed on a plate without hermaphrodites. To start the experiment single males were transferred onto the mating plates with the 14 hermaphrodites. Within 9 hours, the plates were inspected 14 times (after 10 minutes, 1 h, 2 h, 4 h, 4.5 h, 5 h, 5.5 h, 6 h, 6.5 h, 7 h, 7.5 h, 8 h, 8.5 h and 9 h). At every inspection, the male was scored as either having no contact with a hermaphrodite, being in contact with a hermaphrodite, or having its spicule inserted.

### Self-brood-size assay

This assay served to determine the number of offspring produced trough self-fertilization in the absence of males for two natural isolates, N2 and CB4856. Young adult hermaphrodites, which had no developing embryos in the uterus yet, were placed individually onto plates and moved to new plates at least once every day until they stopped laying eggs. Three days after the removal of the mother the progeny on every plate was counted and summed up to total numbers.

### Hermaphrodite outcrossing efficiency assay

This assay was used to test how more frequent mating affects the hermaphrodite's production of self- as well as cross-progeny. Mating plates were prepared four days prior to the assay. One L4 hermaphrodite was placed together with 1, 3, 6 or 12 males onto a mating plate. The hermaphrodite was transferred onto a new mating plate with new young males every day until it stopped laying eggs. The old males were removed from the plates. After one day, additional food bacteria (OP50) were added to the plates and hermaphrodites and males were counted two days later, when they were young adults.

### Statistical analysis

All statistical analyses were done with the program JMP IN version 5.1.2 (SAS Insitute Inc., USA) or SPSS version 14.0 (SPSS Inc., USA). The male proportion over time in populations of the different isolates was evaluated using logistic regression based on a full factorial model with time and either population size or *C. elegans *strain as fixed predictors. The male proportion at specific time points was additionally examined with a Wilcoxon sign rank test. Variation in the number of mated hermaphrodites, the number of offspring per male, and the self- and cross-progeny per mated hermaphrodite was assessed with a General linear model, using an incomplete block design, including male strain, hermaphrodite strain and the interaction between them as fixed factors and experimental block as a random factor. In case of significant interaction terms, significant differences among groups were evaluated with Tukey HSD posthoc tests. Variation in offspring number per repeatedly mated hermaphrodite was tested with an ANOVA, using a full factorial design with male strain and hermaphrodite strain as fixed factors. Subsequent posthoc tests were performed with Tukey HSD. A similar ANOVA was performed to assess the effect of different numbers of males on offspring numbers of repeatedly mated hermaphrodites. In this case, number of males was used as a fixed predictor in the model. With respect to mating behaviour, the time measurements until first contact or first copula were always terminated after 60 min, resulting in non-continuous data. Therefore, differences between crosses were assessed using the Wilcoxon sign rank test. The variation in the number of copulas during this time frame were examined with the Fisher exact test. Differences in the number of contacts or copulations over 14 observation points within a 9 h interval were assessed with the Wilcoxon sign rank test, since the data were non-parametric.

## Results and Discussion

### Variation in male maintenance among *C. elegans *strains

**Figure 1:**
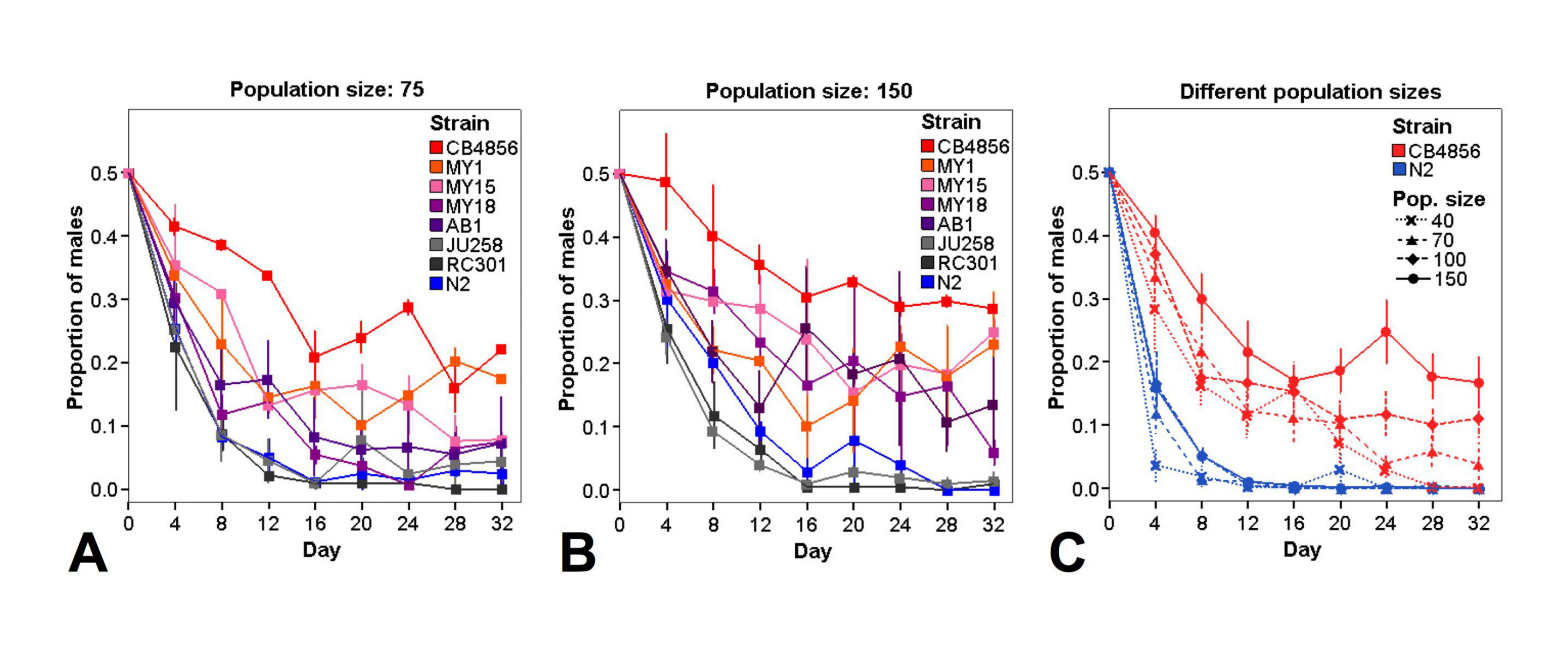
**Persistence of males over time in different *C. elegans *strains and population sizes**. The proportion of males after the indicated number of days is given. Every four days the populations were reduced to the number specified. Error bars are standard errors. All experiments were started with populations containing approximately 50% males. The first actual measurement was done after the first generation at day 4. A) Decrease of the male frequencies in different wild isolates at population size 75. Each point is the average of two independent measurements. B) Decrease of the male frequencies in different wild isolates at population size 150. Each point is the average of two independent measurements. C) Decrease of the male frequencies in four different population sizes in N2 and CB 4856. Each point is the average of five independent measurements. For more details on results and statistical analysis [see additional files 1, 2, 3, 4].

We first tested in how far there is variation in male maintenance among populations of different natural *C. elegans *isolates. Eight strains were tested at two different arbitrarily chosen population sizes (75 and 150). The proportion of males was significantly affected by the factor time, the strain studied, and also the interaction of the two. In particular, males disappeared completely from the cultures of some strains, among them N2. In contrast, in other strains, among them CB4856, the cultures appeared to reach a stable frequency of males after about two weeks (Fig. 1 and [see Additional files 1, 2]).

To refine the analysis we examined the persistence of males at four different arbitrarily chosen population sizes (40, 70, 100 and 150) for N2 and CB4856 (Fig. 1C and [see Additional files 3, 4]). The proportion of males was significantly reduced within few generations. In fact, males disappeared almost entirely from all N2 cultures. The loss was much slower in CB4856 populations. Here, male frequencies were significantly affected by population size, whereby larger populations (100 and 150) sustained a higher number of males [see Additional file 3]. For these two population sizes, the final male frequency was significantly different between N2 and CB4856, while this was not the case for the smaller populations [see Additional file 4].

Taken together, our results confirm long-standing anecdotal knowledge available within the *C. elegans *community: We, and many others, have noticed that it is necessary to deliberately set up crosses with an excess of males every few generations in order to maintain N2 populations with males for genetic analysis. For CB4856 this is not necessary. Our data are also in agreement with previous studies, in which males were rapidly lost in experimental populations of N2 [7,8,10,11] but maintained at constant levels in those of CB4856 [11]. Since during our experiments the different strains were kept in parallel under identical conditions, the difference in male maintenance between them must have a genetic basis. Thus, our results suggest that *C. elegans *bears considerable intra-specific genetic variation that affect male frequency, making it a potentially selectable trait. Interestingly, population size differences had a significant effect on male persistence. This finding may be a consequence of the population size itself, e.g. smaller populations may loose males more often due to chance, thus accelerating male decline. A non-exclusive alternative explanation may be density differences among the population sizes. In this case, higher densities in the large population sizes may associate with more male-hermaphrodite contacts, which could result in higher mating rates, thus stabilizing male frequencies. At the moment, our results do not allow to distinguish between these two effects.

We decided to further characterize the proximate processes that account for variation in male persistence between two of the extremes, namely the strain N2 and CB4856.

### Proximate determinants of male persistence: Male mating efficiency

**Figure 2:**
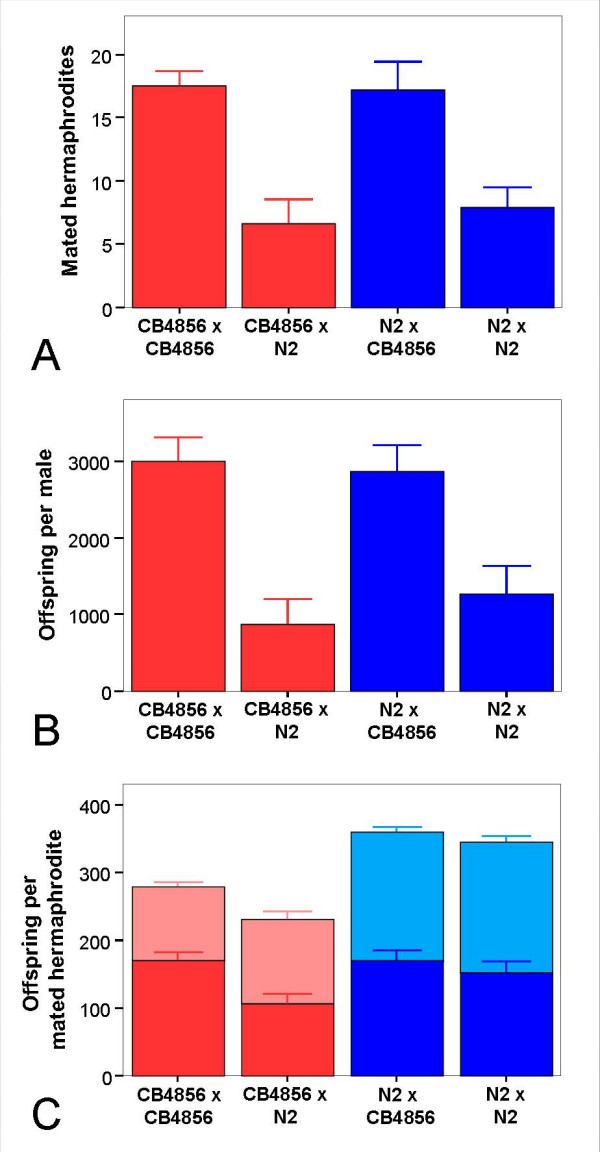
**Mating ability and offspring production in reciprocal crosses between N2 and CB4856**. A single male was crossed with 14 hermaphrodites and was transferred onto a new plate with 14 young hermaphrodites every day for six days. For crosses the hermaphrodite strain is mentioned first and the male strain second. A) Number of successful copulations a male achieved during its life time. B) Total number of cross-progeny produced per male (estimated as twice the number of males). C) Number of cross- (dark color) and self- (light color) progeny produced per successfully mated hermaphrodite after they were separated from the male. Values are the average of five independent replicates. The error bars designate standard errors. Note: C does not include the progeny these hermaphrodites produced during the time they were on the mating plates. For further details see Materials and Methods.

The factors underlying variation in male maintenance were systematically assessed by reciprocal crosses. The analysis was based on all possible mating combinations between CB4856 and N2, using an incomplete block design (i.e. not all combinations were assayed at the same time; see methods). The main experiment focused on the consequences of repeated mating for individual males, which is likely to be realistic in most populations, where male frequency is usually less than 0.01 [24]. Three parameters were simultaneously evaluated: i) The number of successful copulations per individual male, which was offered an excess of virgin hermaphrodites (14 hermaphrodites) every day over a period of six days, ii) the number of sired offspring per male with – accordingly – virtually unlimited access to mates, and iii) the number of cross- and self-progeny per successfully mated hermaphrodite (for experimental details see Methods). Since early reproduction should have a stronger influence on population dynamics than late reproduction, we also performed a separate analysis of the data from only the first two days (Table 1B). The results lead to essentially identical conclusions like the results for the full reproductive period (Figure 2 and Table 1). Therefore, in the following we focus our discussion on the data for the full reproductive life-span.

**Table 1:** Variation in the number of mated hermaphrodites, offspring per male as well as cross- and self-progeny per mated hermaphrodite for the whole experimental period^a^

Cross	Mates	Offspring/male	Cross-progeny/herm	Self-progeny/herm.
			
	Mean ± SE	Mean ± SE	Mean ± SE	Mean ± SE
N2 × N2	7.9 ± 1.6	1272.8 ± 367.7	151.2 ± 16.6	192.2 ± 8.8
N2 × CB4856	17.2 ± 2.2	2860.6 ± 341.5	170.6 ± 13.8	190.7 ± 7.2
CB4856 × N2	6.7 ± 1.9	870.9 ± 335.8	106.4 ± 13.8	124.1 ± 11.3
CB4856 × CB4856	17.5 ± 1.2	3003.2 ± 315.1	170.1 ± 11.4	108.1 ± 7.3

Analysis				
Whole model	*F*_6,32 _= 5.81; *P *<**0.001**	*F*_6,32 _= 4.93; *P *= **0.001**	*F*_6,32 _= 2.31; *P *= 0.058	*F*_6,32 _= 16.2; *P *<**0.001**
Male strain	*F*_1 _= 17.95, *P *<**0.001**	*F*_1 _= 13.67, *P *<**0.001**	*F*_1 _= 3.71, *P *= 0.063	*F*_1 _= 3.41, *P *= 0.074
Hermaphrodite strain	*F*_1 _= 0.23, *P *= 0.636	*F*_1 _= 0.02, *P *= 0.888	*F*_1 _= 0.56, *P *= 0.460	*F*_1 _= 50.53, *P *<**0.001**
Interaction	*F*_1 _= 0.13, *P *= 0.722	*F*_1 _= 0.54, *P *= 0.466	*F*_1 _= 2.42, *P *= 0.130	*F*_1 _= 0.78, *P *= 0.383

*a*, For each cross (top half of the table), the hermaphrodite strain is given first, the male strain last. The mean number of mated hermaphrodites per male, the mean number of offspring per male, the mean number of cross-progeny per male and mated hermaphrodite as well as the mean number of self-progeny per male and mated hermaphrodite are shown. SE, standard error. Statistical results (bottom half of the table) are shown for the whole model. If the latter shows at least a trend (*P *< 0.01), then the statistical importance of different factors in the model are given. The model also included the random factor "experimental date", which however never produced a significant effect (*F*_3 _< 2.7, *P *> 0.06). Significant probabilities are given in bold.

CB4856 males had significantly more successful copulations (Figure 2A and Table 1) and significantly more offspring than N2 males (Figure 2B and Table 1). These two traits were not significantly affected by the hermaphrodite strain used. Therefore, the difference in progeny production by males is most likely a consequence of the differences in mating rates. It does not seem to be caused by variations in the fertilization rates males achieve per successful mating event: The number of cross-progeny per mated hermaphrodite was not significantly affected by any factor of the model or the overall model as a whole (*P *> 0.05; Figure 2C and Table 1). At the same time, it is interesting to note that N2 males appear to produce more cross-progeny with N2 rather than CB4856 hermaphrodites (Fig. 2C; see also similar results obtained after repeated mating of hermaphrodites in Fig. 4A). This effect may account for the trend of a difference produced by the factor male strain in this context (Table 1). It is responsible for the significant interaction term, which was inferred for the data from the first two days (Table 2). One possible explanation for this result is a certain degree of genetic incompatibility, which only becomes visible in one type of cross between the two strains (male N2 and hermaphrodite CB4856) and which may be related to the recent report of genetic incompatibilities among different natural *C. elegans *isolates [33].

**Table 2:** Variation in the number of mated hermaphrodites, offspring per male as well as cross- and self-progeny per mated hermaphrodite for the first two days only^a^

Cross	Mates	Offspring/male	Cross-progeny/herm	Self-progeny/herm.
			
	Mean ± SE	Mean ± SE	Mean ± SE	Mean ± SE
N2 × N2	5.6 ± 1.0	713.0 ± 134.9	136.3 ± 13.2^A,B^	129.4 ± 10.0
N2 × CB4856	11.1 ± 1.3	1283.4 ± 121.0	123.2 ± 12.5^A,B^	157.5 ± 9.7
CB4856 × N2	4.2 ± 0.8	412.4 ± 120.8	84.8 ± 12.0^A^	101.2 ± 13.3
CB4856 × CB4856	9.0 ± 0.5	1192.8 ± 105.8	131.8 ± 9.1^B^	92.4 ± 9.0

Analysis				
Whole model	*F*_6,32 _= 5.95; *P *<**0.001**	*F*_6,32 _= 5.48; *P *<**0.001**	*F*_6,32 _= 2.24; *P *= 0.064	*F*_6,32 _= 6.22; *P *<**0.001**
Male strain	*F*_1 _= 13.88, *P *<**0.001**	*F*_1 _= 12.68, *P *= **0.001**	*F*_1 _= 0.86, *P *= 0.360	*F*_1 _= 0.28, *P *= 0.600
Hermaphrodite strain	*F*_1 _= 0.90, *P *= 0.351	*F*_1 _= 0.47, *P *= 0.499	*F*_1 _= 0.55, *P *= 0.464	*F*_1 _= 10.02, *P *= **0.003**
Interaction	*F*_1 _= 0.19, *P *= 0.668	*F*_1 _= 0.66, *P *= 0.421	*F*_1 _= 6.44, *P *= **0.016**	*F*_1 _= 3.73, *P *= 0.063

*a*, For each cross (top half of the table), the hermaphrodite strain is given first, the male strain last. The mean number of mated hermaphrodites per male, the mean number of offspring per male, the mean number of cross-progeny per male and mated hermaphrodite as well as the mean number of self-progeny per male and mated hermaphrodite are given. SE, standard error. Statistical results (bottom half of the table) are shown for the whole model. If the latter shows at least a trend (*P *< 0.01), then the statistical importance of different factors in the model are given. In case of a significant interaction factor (cross-progeny per mated hermaphrodite), we also provide the results of Tukey HSD posthoc tests, whereby significantly different groups are indicated by different superscript Captial letters in the top part of the table. The model also included the random factor "experimental date", which did not produced a significant effect (*F*_3 _< 1, *P *> 0.4) except of the analysis of self-progeny per mated hermaphrodites (*F*_3 _= 3.03, *P *= 0.044). Significant probabilities are given in bold.

Interestingly, N2 hermaphrodites always had significantly more self-progeny than CB4856 – irrespective of the type of the male and irrespective of the cross-progeny produced (Figure 2C and Table 1). This can be explained by their generally higher fertility. In agreement with other authors [15,34] we found in a separate experiment (self-brood-size assay; see methods) that unmated N2 hermaphrodites produced more self-progeny than unmated CB4856 hermaphrodites (284.3 ± 5.7 and 245.3 ± 6.1, respectively; t-test, *t*_37 _= -4.65, *P *< 0.001). In general, our results on total cross-progeny sired by N2 males (Table 1) as well as total self-progeny by N2 hermaphrodites (see above) are within the range of previously published data [15,34].

### Further dissection of mating behavior

In a separate set of experiments we measured different aspects of mating behavior within either the first one or nine hours. As in the above experiments, we combined an individual male with 14 hermaphrodites, although in this case both were always of the same strain (i. e. 1 N2 male × 14 N2 hemaphrodites and 1 CB4856 male × 14 CB4856 hermaphrodites; for further details see Materials and Methods). Within the first one hour, the different crosses did not show any significant variation, neither regarding the time until first male-hermaphrodite contact (253.3 ± 51.4 sec for N2 and 171.5 ± 28.4 sec for CB4856; Wilcoxon test, *Z *= 0.95, N = 20 per strain, *P *= 0.343), nor in the time until first successful spicule insertions (2148.5 ± 280.6 sec for N2 and 2212 ± 299.7 sec for CB4856; Wilcoxon test, *Z *= 0.10, N = 20 per strain, *P *= 0.923), nor in the number of replicates with successful spicule insertions (12 out of 20 for N2 and 13 out of 20 for CB4856; Fisher exact test, *P *> 0.999). We conclude that the CB4856 males are neither generally better in finding their first mates nor in achieving their first spicule insertion. This result is consistent with the previous finding that N2 and CB4856 show similar behavioral responses to a hermaphrodite-derived cue [12-14].

**Figure 3:**
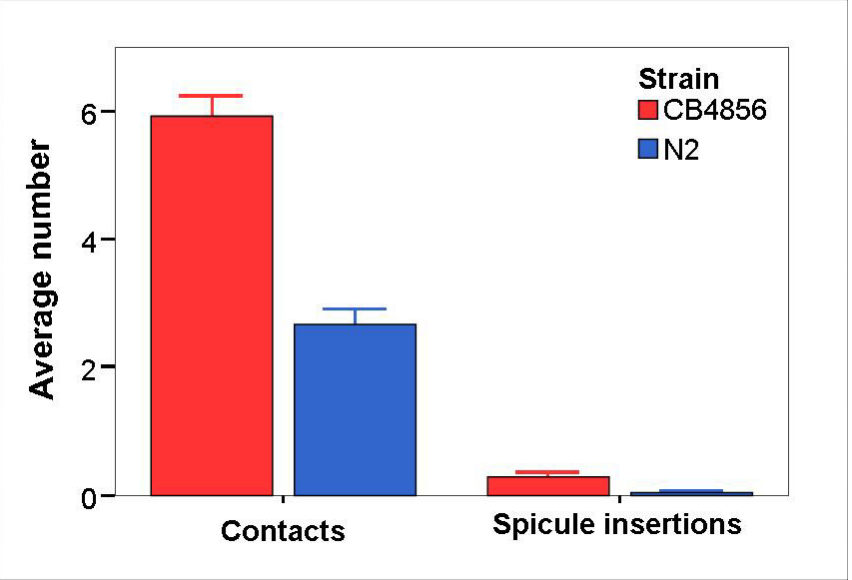
**Number of contacts and spicule insertions observed over 14 observation points within 9 hours**. Mating assays with one male and 14 hermaphrodites were set up for CB4856 and N2, using 45 and 47 replicates, respectively. The plates were inspected 14 times within the first 9 hours. The figure shows the average number of male-hermaphrodite contacts and spicule insertions. Each spicule insertion was also considered to be a contact. The error bars designate standard errors. For exact numbers and statistical analysis [see additional file 5].

However, over 14 observation points within the first 9 hours, the CB4856 × CB4856 crosses produced significantly more male-hermaphrodite contacts (Fig. 3 and [see Additional file 5]; Wilcoxon test, *Z *= 6.59, N = 45 for CB4856, N = 47 for N2, *P *< 0.001) and spicule insertions (Fig. 3 and [see Additional file 5]; Wilcoxon test, *Z *= 2.98, N = 45 for CB4856, N = 47 for N2, *P *= 0.003). From these observations, we conclude that over time CB4856 males achieve a higher rate of mate contacts and spicule insertions than N2 males. These results are in excellent agreement with the results presented in Fig. 2 and Table 1. Consequently, an overall higher mating frequency could contribute to the observed higher male persistence in CB4856 relative to N2. We cannot explain why N2 males mate less frequently than CB4856. One possibility would be that N2 males require a longer time to replenish their sperm stocks. If so, they could produce a smaller number of sperm over their life time which would explain the reduced number of progeny sired.

Variation in male sperm competitiveness (size) could have also affected the observed differences in male persistence. We have not as yet measured this trait. However it is unlikely to have significantly influenced the results on mating efficiency in Fig. 2. We followed the mated hermaphrodites until they ceased to produce progeny, presumably because they had used up their supply of sperm (including both male and hermaphrodite sperm). In this case, all male sperm transferred during mating should have contributed to offspring production irrespective of their competitiveness.

### Consequence of repeated mating of hermaphrodites

In the experiments leading to Figure 2 and Table 1 it is likely that individual hermaphrodites mated only once or very few times since they were in excess and they were removed from the males after one day. Given the low number of males in natural *C. elegans *populations, mating only once, if at all, might be realistic. However, in our male maintenance assays (see Figure 1), the male frequency was initially set to approximately 0.5, thus allowing for repeated mating interactions. Therefore, we asked whether repeated mating could influence the number of self- as well as cross-progeny. For this purpose, we set up all reciprocal crosses between N2 and CB4856 and, for each cross, single hermaphrodites were mated each day with a virgin male until the hermaphrodite ceased to produce progeny (see Materials and Methods, hermaphrodite outcrossing efficiency assay with one male).

**Figure 4:**
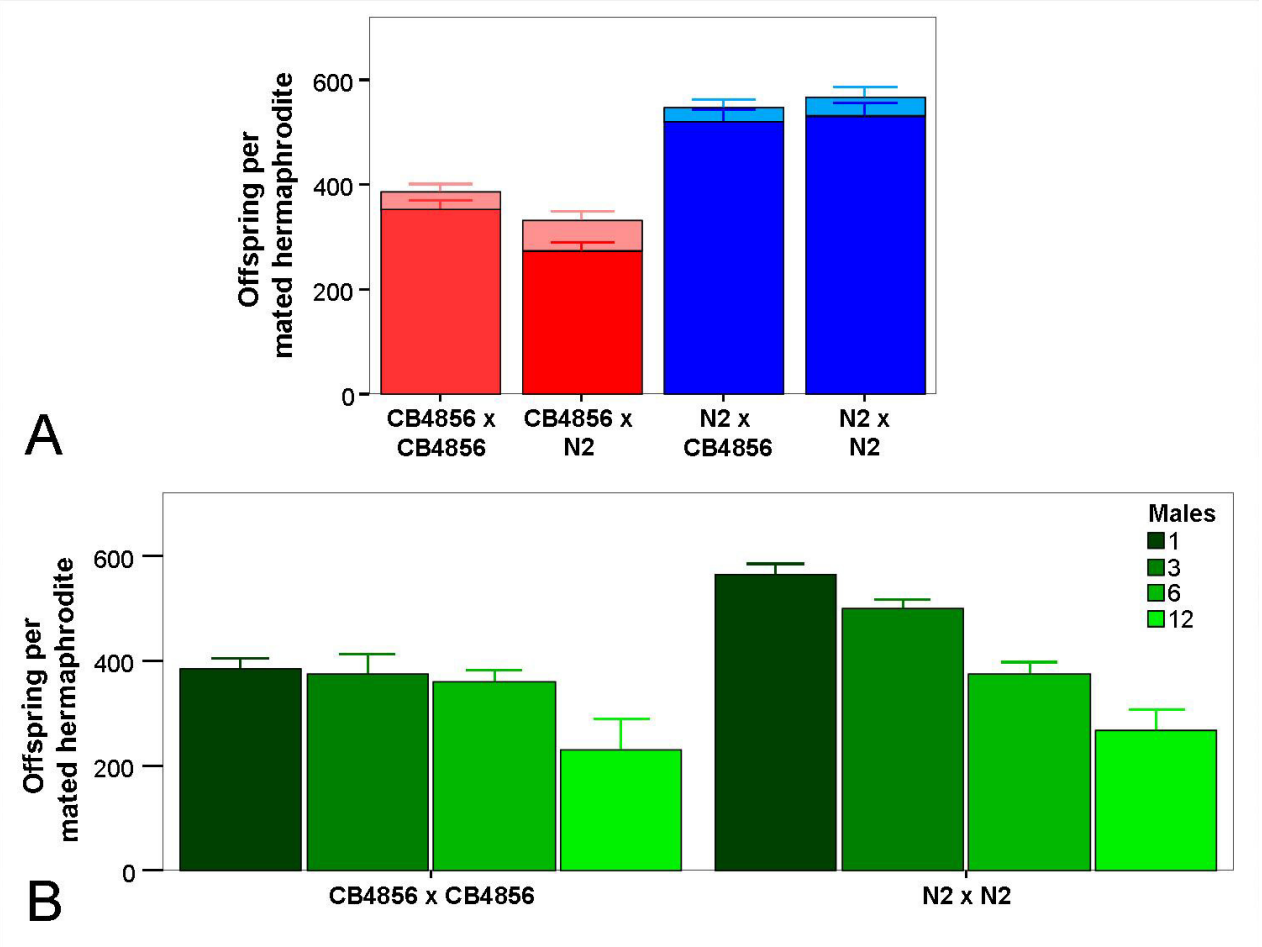
**Offspring production by repeatedly mated hermaphrodites**. A) Repeated mating of an individual hermaphrodite with a single virgin male every day. The hermaphrodite strain is indicated first and the male strain second. The number of cross-progeny (dark colors) and self-progeny (light colors) were determined. Each value is the average of five replicates. The error bars represent standard errors. For exact numbers and statistical analysis [see additional file 7]. Repeated mating of individual hermaphrodites with different numbers of virgin males added every day. The figure shows total progeny. Note that the proportion of self-progeny was low (see A) and did not differ significantly between treatments; thus, the observed variation is mainly determined by cross-progeny. Each value is the average of five hermaphrodites. The error bars represent standard errors. For exact numbers and statistical analysis [see additional file 8].

In agreement with earlier literature [34], we found that repeated mating (repeated mating assay with one male, see Methods) increased the total number of progeny and the proportion of cross-progeny (Figure 4A and [see Additional file 6]). N2 hermaphrodites produced significantly more progeny than CB4856 hermaphrodites (ANOVA, hermaphrodite strain effect, *F*_1 _= 107.69, *P *< 0.001) and the proportion of self-progeny was small. The origin of the male did not make any significant difference (ANOVA, male strain effect, *F*_1 _= 2.93, *P *= 0.108). Analysis of the first two days only produced essentially identical results [see Additional file 7]. Consequently, in the case of repeated mating of hermaphrodites, N2 would produce a significantly larger number of cross-progeny and thus a larger proportion of males than CB4856.

At first sight, this result appears to contradict our findings from the male maintenance assays, where N2 populations loose males rapidly in contrast to CB4856. However, hermaphrodites in the repeated mating experiment were exposed to "new" virgin males every day (most likely with high mating efficiency), whereas hermaphrodites in the male maintenance assay encounter the same males over consecutive days, which are likely to show reduced mating efficiency over time (due to the likely high energy demand per mating as well as sperm depletion). The reduction in mating efficiency is particularly pronounced in N2 (see results from the mating behaviour experiments above). Therefore, we expect fewer matings, a relatively larger number of self-progeny, and thus a continuous decrease of males in the N2 populations over time.

A possible higher cost of multiple mating may further contribute to more rapid initial male decline in N2 in the male maintenance assays. Repeated mating with increasing numbers of males caused a significant reduction in offspring number in both strains (Figure 4B and [see Additional file 8]). Importantly, this reduction was more pronounced for N2, for which it caused a loss of up to 53% progeny compared to a maximum of about 40% for CB4856 (comparison between repeated mating with 1 versus 12 males). Consequently, if repeated mating of hermaphrodites did occur in the experimental populations, then the higher costs (i.e. offspring loss) of copulations with multiple males for N2 should decrease male frequencies and thus outcrossing rates to a larger extent in N2 than CB4856.

The finding of a cost of multiple mating is likely a consequence of sexual conflict, as reported for a large diversity of organisms [35,36]. One possible explanation for this observation could be increased intra-sexual male-male competition or otherwise detrimental male-male interactions, which were shown in the past to decrease *C. elegans *male life-span [37]. Alternatively, it could result from inter-sexual antagonisms such as those mediated by male manipulative substances that are transferred during copulation, in order to enhance male fertilization success [35,36]. In *C. elegans*, the possible relevance of such inter-sexual conflict was previously indicated by reduced hermaphrodite longevity after mating [38].

## Conclusion

Our experiments suggest that the combination of two traits are likely involved in determining the difference in male maintenance between CB4856 and N2: i) CB4856 males achieved a larger number of successful copulations and therefore sired more cross-progeny than N2. Consequently, a mixed-gender CB4856 population will contain a larger number of cross-fertilized hermaphrodites and thus produce more males than a corresponding N2 population. The resulting higher frequency of males should further enhance cross-fertilization rates in CB4856, because male density positively links with hermaphrodite mating rates [9]. ii) Unmated and singly mated N2 hermaphrodites produced a higher number of self-progeny than corresponding CB4856 hermaphrodites. This parameter reduces male density and thus mating rates in N2, which additionally amplifies the loss of males within N2 populations.

## Authors' contributions

VW did all the bench work and participated in the experimental design, the analysis of the data and the writing of the manuscript. HS participated in the experimental design and the data analysis. He did all the statistical analyses and he co-wrote the manuscript together with AS. AS participated in the experimental design and the data analysis. He coordinated the whole work and supervised the practical work. He co-wrote the manuscript together with HS. The contributions of HS and AS should be considered equal. All Authors read and approved the final manuscript.

## Supplementary Material

Additional file 1 Supplementary table 1. Logistic regression of male proportion in different natural isolates. Click here for file

Additional file 2 Supplementary table 2. Male proportion averaged over days 16 to 32 for different strains and two population sizes. Click here for file

Additional file 3 Supplementary table 3. Logistic regression of male proportion in N2 and CB4856 with different population sizes. Click here for file

Additional file 4 Supplementary table. Male proportion on day 32 for different population sizes of the strains N2 and CB4856. Click here for file

Additional file 5 Supplementary table 5. Variation in the number of contacts and spicule insertions within the first 9 hours. Click here for file

Additional file 6 Supplementary table 6. Variation in the number of cross- and self-progeny per repeatedly mated hermaphrodite for the whole experimental period. Click here for file

Additional file 7 Supplementary table 7. Variation in the number of cross- and self-progeny per repeatedly mated hermaphrodite for the first two days only. Click here for file

Additional file 8 Supplementary table 8. Variation in total hermaphrodite offspring number after repeated mating to either 1, 3, 6, or 12 males. Click here for file
