# Supplementary material for: Experimental insight into the proximate causes of male persistence variation among two strains of the androdioecious Caenorhabditis elegans (Nematoda)
Source: BMC Ecol. 2008 Jul 13;8:12. doi: 10.1186/1472-6785-8-12 (PMC2483263; doi:10.1186/1472-6785-8-12)
Supplement: Additional file 1 — Supplementary table 1. Logistic regression of male proportion in different natural isolates. [file 1472-6785-8-12-S1.doc]

Supplementary table 1: Logistic regression of male proportion in different natural isolatesa

| Effect | All days |  |  |  | Days 16-32 | |  |
| --- | --- | --- | --- | --- | --- | --- | --- |
|  | *χ^2^* | df | *P* |  | *χ^2^* | df | *P* |
| Population size: 75 |  |  |  |  |  |  |  |
| Strain | 75.08 | 7 | **<0.001** |  | 55.03 | 7 | **<0.001** |
| Day | 97.35 | 1 | **<0.001** |  | 0.03 | 1 | 0.860 |
| Strain x day | 23.51 | 7 | **0.001** |  | 19.62 | 7 | **0.006** |
| Replicate | 31.51 | 8 | **<0.001** |  | 39.09 | 8 | **<0.001** |
| Population size: 150 |  |  |  |  |  |  |  |
| Strain | 75.72 | 7 | **<0.001** |  | 56.60 | 7 | **<0.001** |
| Day | 106.50 | 1 | **<0.001** |  | 3.39 | 1 | 0.066 |
| Strain x day | 40.18 | 7 | **<0.001** |  | 20.56 | 7 | **0.005** |
| Replicate | 21.28 | 8 | **0.006** |  | 26.57 | 8 | **0.001** |

a, For both strains, the used regression model explained a significant part of the variance (*P* <0.001) and provided a good fit to the data (*P* >0.999). The importance of the different factors were assessed with an Effect Wald test. The above results suggest that from day 16 onwards the male proportion does not change any more over time (insignificant value for factor Day in left part of table) and thus is likely to have reached equilibrium. Therefore, we combined them for subsequent pairwise comparisons (Supplementary table 1B below).
