# Supplementary material for: Experimental insight into the proximate causes of male persistence variation among two strains of the androdioecious Caenorhabditis elegans (Nematoda)
Source: BMC Ecol. 2008 Jul 13;8:12. doi: 10.1186/1472-6785-8-12 (PMC2483263; doi:10.1186/1472-6785-8-12)
Supplement: Additional file 2 — Supplementary table 2. Male proportion averaged over days 16 to 32 for different strains and two population sizes. [file 1472-6785-8-12-S2.doc]

Supplementary table 2: Male proportion averaged over days 16 to 32 for different strains and two population sizesa

| Strain | Male proportion | Δ N2 |  |  | Δ CB4856 |  |
| --- | --- | --- | --- | --- | --- | --- |
|  | Mean ± SE | *Z* | *P* |  | *Z* | *P* |
| Population size: 75 | |  |  |  |  |  |
| N2 | 0.021 ± 0.006 | n.a. |  |  | -3.75 | **<0.001**** |
| CB4856 | 0.224 ± 0.017 | -3.75 | **<0.001**** |  | n.a. |  |
| MY1 | 0.159 ± 0.015 | -3.18 | **0.002**** |  | -2.34 | **0.019**** |
| MY15 | 0.122 ± 0.017 | -2.93 | **0.003**** |  | -2.39 | **0.017**** |
| MY18 | 0.048 ± 0.011 | -1.38 | 0.168 |  | -3.34 | **<0.001**** |
| AB1 | 0.068 ± 0.019 | -1.63 | 0.103 |  | 3.02 | **0.003**** |
| JU258 | 0.039 ± 0.016 | -0.14 | 0.886 |  | -3.56 | **<0.001**** |
| RC301 | 0.006 ± 0.003 | -0.86 | 0.388 |  | -3.70 | **<0.001**** |
| Population size: 150 | |  |  |  |  |  |
| N2 | 0.030 ± 0.017 | n.a. |  |  | -3.79 | **<0.001**** |
| CB4856 | 0.302 ± 0.006 | -3.79 | **<0.001**** |  | n.a. |  |
| MY1 | 0.176 ± 0.030 | -2.30 | **0.021**** |  | -3.14 | **0.002**** |
| MY15 | 0.205 ± 0.024 | -2.87 | **0.004**** |  | -2.63 | **0.009**** |
| MY18 | 0.149 ± 0.031 | -2.19 | **0.029**** |  | -2.66 | **0.008**** |
| AB1 | 0.178 ± 0.034 | -2.09 | **0.036**** |  | 2.80 | **0.005**** |
| JU258 | 0.017 ± 0.034 | 0.10 | 0.924 |  | -4.10 | **<0.001**** |
| RC301 | 0.005 ± 0.002 | -0.47 | 0.641 |  | -3.85 | **<0.001**** |

*a*, The difference to either N2 or CB4856 was examined with the Wilcoxon sign rank test (N = 10 for each strain). Significance is indicated by * for α ≤ 0.05 according to the false discovery rate; the corresponding significant probabilities are given in bold.
