# Supplementary material for: Experimental insight into the proximate causes of male persistence variation among two strains of the androdioecious Caenorhabditis elegans (Nematoda)
Source: BMC Ecol. 2008 Jul 13;8:12. doi: 10.1186/1472-6785-8-12 (PMC2483263; doi:10.1186/1472-6785-8-12)
Supplement: Additional file 3 — Supplementary table 3. Logistic regression of male proportion in N2 and CB4856 with different population sizes. [file 1472-6785-8-12-S3.doc]

Supplementary table 3: Logistic regression of male proportion in N2 and CB4856 with different population sizes^a^

| Effect | *χ^2^* | df | *P* |
| --- | --- | --- | --- |
| Strain: N2 |  |  |  |
| Population size | 3.24 | 3 | 0.356 |
| Day | 90.48 | 1 | **<0.001** |
| Population size x day | 6.32 | 3 | 0.097 |
| Replicate | 20.19 | 16 | 0.212 |
| Strain: CB4856 |  |  |  |
| Population size | 13.85 | 3 | **0.003** |
| Day | 152.13 | 1 | **<0.001** |
| Population size x day | 43.25 | 3 | **<0.001** |
| Replicate | 54.16 | 16 | **<0.001** |

a, For both strains, the used regression model explained a significant part of the variance (*P* <0.001) and provided a good fit to the data (*P* >0.999). The importance of the different factors were assessed with an Effect Wald test. Significant probabilities are given in bold.
