# Supplementary material for: Experimental insight into the proximate causes of male persistence variation among two strains of the androdioecious Caenorhabditis elegans (Nematoda)
Source: BMC Ecol. 2008 Jul 13;8:12. doi: 10.1186/1472-6785-8-12 (PMC2483263; doi:10.1186/1472-6785-8-12)
Supplement: Additional file 4 — Supplementary table. Male proportion on day 32 for different population sizes of the strains N2 and CB4856. [file 1472-6785-8-12-S4.doc]

Supplementary table 4: Male proportion on day 32 for different population sizes of the strains N2 and CB4856^a^

| Pop. size | N2 | CB4856 | *Z* | *P* |
| --- | --- | --- | --- | --- |
|  | Mean ± SE | Mean ± SE |  |  |
| 40 | 0 ± 0 | 0.001 ± 0.001 | -0.80 | 0.424 |
| 70 | 0 ± 0 | 0.038 ± 0.036 | -1.34 | 0.180 |
| 100 | 0 ± 0 | 0.111 ± 0.031 | -2.67 | **0.008** |
| 150 | 0 ± 0 | 0.167 ± 0.041 | -2.67 | **0.008** |

*a*, The difference between N2 and CB4856 was assessed with a Wilcoxon sign rank test (N = 5 for each strain and population size). Significant probabilities are given in bold. Comparisons were only made for the last measurement date, because for CB4856 some of the population sizes did not reach equilibrium values over the last couple of transfer dates (significant effect for the factor Day; results not shown). Essentially identical results are obtained if the above comparison is performed for the three earlier transfer days.
