# Supplementary material for: Experimental insight into the proximate causes of male persistence variation among two strains of the androdioecious Caenorhabditis elegans (Nematoda)
Source: BMC Ecol. 2008 Jul 13;8:12. doi: 10.1186/1472-6785-8-12 (PMC2483263; doi:10.1186/1472-6785-8-12)
Supplement: Additional file 5 — Supplementary table 5. Variation in the number of contacts and spicule insertions within the first 9 hours. [file 1472-6785-8-12-S5.doc]

Supplementary table 5: Variation in the number of contacts and spicule insertions within the first 9 hours^a^

| Cross (top) | Contacts | Spicule insertions |
| --- | --- | --- |
| Analysis (bottom) | Mean ± SE | Mean ± SE |
| N2 x N2 | 2.66 ± 0.24 | 0.04 ± 0.03 |
| CB4856 x CB4856 | 5.93 ± 0.30 | 0.29 ± 0.08 |
| Analysis |  |  |
| *Z* | 6.59 | 2.98 |
| *P* | < **0.001** | **0.003** |

*a*, The values give the mean number of contacts or spicule insertions over 14 observation points within a 9 h period. The difference between crosses was assessed with a Wilcoxon sign rank test (N = 47 for N2 x N2 and N = 45 for CB4856 x CB4856). Significant probabilities are given in bold.
