# Supplementary material for: Experimental insight into the proximate causes of male persistence variation among two strains of the androdioecious Caenorhabditis elegans (Nematoda)
Source: BMC Ecol. 2008 Jul 13;8:12. doi: 10.1186/1472-6785-8-12 (PMC2483263; doi:10.1186/1472-6785-8-12)
Supplement: Additional file 6 — Supplementary table 6. Variation in the number of cross- and self-progeny per repeatedly mated hermaphrodite for the whole experimental period. [file 1472-6785-8-12-S6.doc]

Supplementary table 6: Variation in the number of cross- and self-progeny per repeatedly mated hermaphrodite for the whole experimental period^a^

| Cross (top) | Cross-progeny | Self-progeny |
| --- | --- | --- |
| Analysis (bottom) | Mean ± SE | Mean ± SE |
| N2 x N2 | 530.4 ± 25.1^A^ | 36.4 ± 19.0 |
| N2 x CB4856 | 520.5 ± 20.6^A^ | 28.5 ± 15.3 |
| CB4856 x N2 | 271.6 ± 16.8^B^ | 58.6 ± 16.4 |
| CB4856 x CB4856 | 352 ± 18.1^B^ | 35.4 ± 14.9 |
| Analysis |  |  |
| Whole model | *F*_3,15_ = 39.05; *P* < **0.001** | *F*_3,15_ = 0.61; *P* = 0.611 |
| Male strain | *F*_1_ = 2.93, *P* = 0.108 |  |
| Hermaphrodite strain | *F*_1_ = 107.69, *P* < **0.001** |  |
| Interaction | *F*_1_ = 4.81, *P* = **0.045** |  |

*a*, For each cross (top half of the table), the hermaphrodite strain is given first, the male strain last. The mean number of cross-progeny and self-progeny per repeatedly mated hermaphrodite are presented. SE, standard error. Statistical results (bottom half of the table) are shown for the whole model. If the latter shows at least a trend (*P* < 0.01), then the statistical importance of different factors in the model are given. In case of a significant interaction term, we also provide the results of Tukey HSD posthoc tests, whereby significantly different groups are indicated by different superscript Capital letters in the top part of the table. Significant probabilities are given in bold.
