# Supplementary material for: Experimental insight into the proximate causes of male persistence variation among two strains of the androdioecious Caenorhabditis elegans (Nematoda)
Source: BMC Ecol. 2008 Jul 13;8:12. doi: 10.1186/1472-6785-8-12 (PMC2483263; doi:10.1186/1472-6785-8-12)
Supplement: Additional file 7 — Supplementary table 7. Variation in the number of cross- and self-progeny per repeatedly mated hermaphrodite for the first two days only. [file 1472-6785-8-12-S7.doc]

Supplementary table 7: Variation in the number of cross- and self-progeny per repeatedly mated hermaphrodite for the first two days only^a^

| Cross/Analysis | Cross-progeny | Self-progeny |
| --- | --- | --- |
|  | Mean ± SE | Mean ± SE |
| N2 x N2 | 178.4 ± 8.7 | 15.6 ± 8.6 |
| N2 x CB4856 | 155.5 ± 13.9 | 34.5 ± 5.9 |
| CB4856 x N2 | 122 ± 16.2 | 39.4 ± 17.2 |
| CB4856 x CB4856 | 132 ± 18.2 | 19 ± 9.7 |
| Analysis |  |  |
| Whole model | *F*_3,15_ = 3.02; *P* = 0.063 | *F*_3,15_ = 1.02; *P* = 0.414 |
| Male strain | *F*_1_ = 0.19, *P* = 0.671 |  |
| Hermaphrodite strain | *F*_1_ = 7.19, *P* = **0.017** |  |
| Interaction | *F*_1_ = 1.22, *P* = 0.287 |  |

*a*, For each cross (top half of the table), the hermaphrodite strain is given first, the male strain last. The mean number of cross-progeny and self-progeny per repeatedly mated hermaphrodite are shown. SE, standard error. Statistical results (bottom half of the table) are shown for the whole model. If the latter shows at least a trend (*P* < 0.01), then the statistical effect of different factors in the model are given. Significant probabilities are given in bold.
