# Supplementary material for: Experimental insight into the proximate causes of male persistence variation among two strains of the androdioecious Caenorhabditis elegans (Nematoda)
Source: BMC Ecol. 2008 Jul 13;8:12. doi: 10.1186/1472-6785-8-12 (PMC2483263; doi:10.1186/1472-6785-8-12)
Supplement: Additional file 8 — Supplementary table 8. Variation in total hermaphrodite offspring number after repeated mating to either 1, 3, 6, or 12 males. [file 1472-6785-8-12-S8.doc]

Supplementary table 8: Variation in total hermaphrodite offspring number after repeated mating to either 1, 3, 6, or 12 males^a^

| Cross | N males | Total Offspring | ANOVA |  |
| --- | --- | --- | --- | --- |
|  |  | Mean ± SE | *F*_3,15_ | *P* |
| N2 x N2 | 1 | 566.8 ± 19.7 | 23.94 | **< 0.001** |
|  | 3 | 500.2 ± 18.8 |  |  |
|  | 6 | 375.6 ± 24.1 |  |  |
|  | 12 | 268.8 ± 40.1 |  |  |
| CB4856 x CB4856 | 1 | 387.4 ± 18.5 | 3.73 | **0.035** |
|  | 3 | 376.0 ± 37.5 |  |  |
|  | 6 | 361.2 ± 21.7 |  |  |
|  | 12 | 230.4 ± 60.1 |  |  |

*a*, The mean number of total progeny per repeatedly mated hermaphrodite was measured over the whole experimental period. The analysis of variance tested an effect of the number of males on total offspring. Significant probabilities are given in bold.
